# Update on the Use of Transcranial Electrical Brain Stimulation to Manage Acute and Chronic COVID-19 Symptoms

**DOI:** 10.3389/fnhum.2020.595567

**Published:** 2020-11-12

**Authors:** Giuseppina Pilloni, Marom Bikson, Bashar W. Badran, Mark S. George, Steven A. Kautz, Alexandre Hideki Okano, Abrahão Fontes Baptista, Leigh E. Charvet

**Affiliations:** ^1^Department of Neurology, NYU Langone Health, New York, NY, United States; ^2^Department of Biomedical Engineering, The City College of New York, New York, NY, United States; ^3^Department of Psychiatry, Medical University of South Carolina, Charleston, SC, United States; ^4^Ralph H Johnson Veterans Affairs Medical Center, Charleston, SC, United States; ^5^Department of Health Sciences and Research, Medical University of South Carolina, Charleston, SC, United States; ^6^Center for Mathematics, Computation and Cognition, Universidade Federal do ABC, São Bernardo do Campo, Brazil; ^7^Brazilian Institute of Neuroscience and Neurothechnology 52 (BRAINN/CEPID53 FAPESP), University of Campinas, Campinas, Brazil; ^8^Laboratory of Medical Investigation 54 (LIM-54), São Paulo University, São Paulo, Brazil

**Keywords:** mental health, neuropsychiatric, TMS, COVID-19, tDCS, TES, NEUROCOVID

## Abstract

The coronavirus disease 19 (COVID-19) pandemic has resulted in the urgent need to develop and deploy treatment approaches that can minimize mortality and morbidity. As infection, resulting illness, and the often prolonged recovery period continue to be characterized, therapeutic roles for transcranial electrical stimulation (tES) have emerged as promising non-pharmacological interventions. tES techniques have established therapeutic potential for managing a range of conditions relevant to COVID-19 illness and recovery, and may further be relevant for the general management of increased mental health problems during this time. Furthermore, these tES techniques can be inexpensive, portable, and allow for trained self-administration. Here, we summarize the rationale for using tES techniques, specifically transcranial Direct Current Stimulation (tDCS), across the COVID-19 clinical course, and index ongoing efforts to evaluate the inclusion of tES optimal clinical care.

## Introduction

Severe acute respiratory syndrome coronavirus 2 (SARS-CoV-2) is a highly infectious virus that has resulted in a global pandemic of coronavirus disease 19 (COVID-19). Over the course of the pandemic, understanding is evolving regarding the nature and course of COVID-19 (SARS-CoV-2) illness as well as its optimal management.

Neuromodulation, which spans a broad range of implanted and non-invasive modalities, may have a potential role in the treatment of COVID-19 related symptoms. This potential is theorized based on the known mechanisms of biological action, demonstrated benefits in non-COVID-19 patients for various known sequelae of COVID-19 illness and recovery (Lefaucheur et al., 2017; David et al., 2018; Ghosh, 2019; Razza et al., 2020), with initial reports of its application in COVID-19 patients (Azabou et al., 2020; Bonaz et al., 2020; Kaniusas et al., 2020; Shinjo et al., 2020; Zhang et al., 2020).

The majority of current investigational efforts for the use of non-invasive stimulation approaches in COVID-19 are focused on managing acute infection through the modification of immunological response and to restore respiratory function via vagus nerve stimulation (VNS; Staats et al., 2020). VNS techniques include invasive and non-invasive manual or electrical (transcutaneous) stimulation, with effects of therapeutic relevance to COVID-19 under clinical investigation (Staats et al., 2020).

This update provides a summary of current efforts in the evaluation and application of non-invasive brain stimulation (NIBS) techniques as interventions in the context of recovery from COVID-19, focusing on transcranial Electrical Stimulation (tES) approaches, and particularly transcranial Direct Current Stimulation (tDCS; Woods et al., 2016) as well as transcranial Alternating Current Stimulation (tACS; Antal et al., 2017). We also note related uses of Transcranial Magnetic Stimulation (TMS; Huang et al., 2009), informing tES applications. However, of amplified importance during the COVID-19 pandemic, tES has the advantage of remote treatment delivery that can be easily scaled (Bikson et al., 2020; Charvet et al., 2020).

tES/TMS techniques are designed to modulate the activity of intracranial brain structures and neural circuitry (Peterchev et al., 2012). These NIBS approaches induce different electric field patterns (Polanía et al., 2018), with tES less focalized and more limited in reaching deep brain structures (Miranda et al., 2013). While tES techniques sometimes involve stimulating ancillary peripheral or cranial nerves [e.g., Adair et al., 2020], this review is concerned only with direct non-invasive brain stimulation.

There are multiple pathways by which tES may address immediate and long-term COVID-19 morbidity, but subject to direct experimental testing in COVID-19 patients these links remain indirect. There is a bidirectional influence between the brain and immune response (Dantzer, 2018). Deep brainstem and forebrain regions mediate the immune response throughout the body and can be potential targets for non-invasive neuromodulatory approaches. While it is impractical to activate deep regions selectivity (e.g., without activating superficial cerebral cortex) using tES, deep brain regions can certainly be reached by the electrical current (Dasilva et al., 2012; Huang et al., 2017; Gomez-Tames et al., 2020; Huang and Zhao, 2020) as well as through axonal connections between cortical activated areas (Bestmann et al., 2005; Bestmann and Feredoes, 2013). The cortical regions conventionally targeted with tES, such as frontal (Iseger et al., 2020) and temporal regions (Montenegro et al., 2011), can be used to influence the systemic immune response and prevent neuroinflammation (Dantzer, 2018). Furthermore, tES may be investigated for application in the restoration of respiratory and musculoskeletal functions during recovery (Vandermeeren et al., 2010).

**FIGURE 1:**
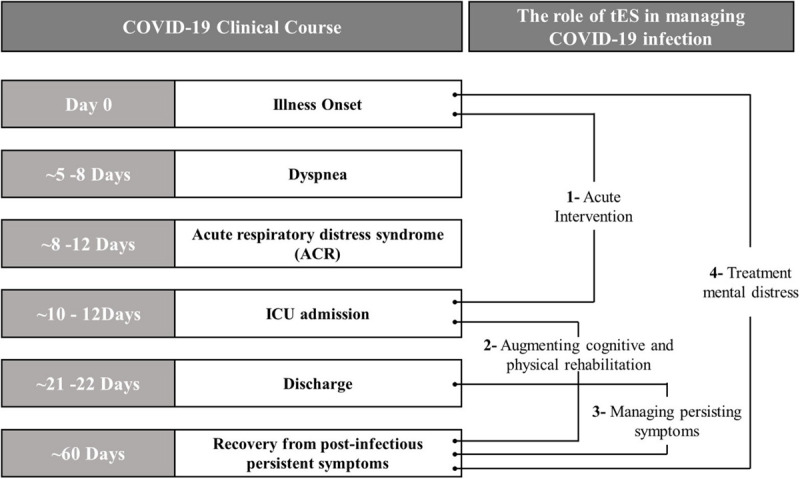
Possible application of tES techniques in the context of COVID-19 clinical course.

Transcranial Electrical Stimulation has the broad potential to manage COVID-19 infection, its complications and related symptoms through four pathways (Figure 1):

1. Acute intervention directly mitigating the infection through the stimulation of regions involved in the regulation of systemic anti-inflammatory responses and/or autonomic responses and prevention of neuroinflammation and recovery of respiration (Rueger et al., 2012; Azabou et al., 2020; Fudim et al., 2020);
2. Add-on treatment to augment cognitive and physical rehabilitation following critical illness (Simpson and Robinson, 2020), as well as treating acute psychological reactions (Holmes et al., 2020);
3. Managing persistent post-infectious symptoms such as fatigue and pain (Baptista et al., 2020);
4. Treatment of outbreak related mental distress including neurological and psychiatric disorders exacerbated by surrounding psychosocial stressors related to COVID-19 (Baptista et al., 2020; Castelo-Branco and Fregni, 2020).

### Potential Role in Acute Infection and Illness

tES approaches are targeted to have direct effects on brain functions which can include modulating perfusion (Zheng et al., 2011; Stagg et al., 2013; Shin et al., 2020), clearance mechanisms (Cancel et al., 2018), and the immune response (Rabenstein et al., 2019). tES, from a theoretical perspective, may be applicable for preventing acute neuroinflammation or to directly address the neurological manifestations of COVID-19 infection.

In addition to the more widely characterized systemic pulmonary and cardiovascular effects (Asadi-Pooya and Simani, 2020), direct CNS involvement and neurologic outcomes from COVID-19 and its treatment are emerging as an important focus (Helms et al., 2020a). For example, in a large Spanish registry, 57% of hospitalized patients had at least one neurological symptom.

With acute infection, COVID-19 commonly presents with CNS symptoms such as headache, anosmia, ageusia, and dizziness (Romero-Sánchez et al., 2020; Zubair et al., 2020). It is possible that the virus is neurotropic (Asadi-Pooya and Simani, 2020). Neuroinvasion, particularly via brainstem involvement, may be directly linked to the respiratory failure syndrome (Li Y.-C. et al., 2020). A sham-controlled RCT (initiated in 2018, pre-COVID-19 pandemic) is testing the efficacy of tDCS over the primary cortical motor area on modulating the excitability of the respiratory neurological pathways for relieving dyspnea in patients admitted to mechanical ventilation in ICU (Azabou et al., 2020).

Additionally, the systemic inflammation in COVID-19 has led to neurologic outcomes including impaired consciousness, delirium, encephalopathy, psychosis, cerebrovascular events, seizure, Guillain-Barré syndrome, and optic neuritis (Helms et al., 2020b; Mao et al., 2020; Rogers et al., 2020; Varatharaj et al., 2020).

There is also a theoretical potential role for tDCS in the prevention and treatment of thrombosis through the modulation of the autonomic nervous system (Montenegro et al., 2011; Schestatsky et al., 2013; Okano et al., 2015). Those patients treated in the ICU have increased risk of microvascular thrombosis, venous thrombosis or arterial thrombosis associated with higher mortality (Fraissé et al., 2020) which demonstrates the significance of coagulation abnormalities in this population. Anticoagulant treatment of thrombosis with low molecular weight heparin tends to be associated with improved prognosis in extreme COVID-19 patients who meet sepsis-induced coagulopathy requirements or with significantly elevated D-dimer (Tang, 2020). Heparins are likely to be a potential approach in the care of COVID−19 patients as they combat hypoxia and generalized organ failure with coagulopathies and because they are likely to reduce cardiovascular arrhythmias and sudden deaths, both associated with COVID−19 itself and its pharmacology (Menezes-Rodrigues et al., 2020). Interestingly, increased sympathetic activity is related to the development of thrombosis (Brook and Julius, 2000), and sympathetic block was also suggested as a potential approach to treatment for thrombosis. Therefore, autonomic nervous modulation has been used as a complementary therapeutic strategy for thrombosis (Soni et al., 2012; Schestatsky et al., 2013; Taheri et al., 2019; Wang et al., 2019).

### Role in Post-acute Recovery of Function

Neurological consequences often result from the management of severe COVID-19 illness, for instance following intubation (Guo et al., 2020; Helms et al., 2020a; Needham et al., 2020). COVID-19 infection can cause serious injury to cranial nerves and peripheral nerves (Fotuhi et al., 2020), resulting in muscle weakness, muscle injuries, facial paresis, sensory ataxia, flaccid diplegia, or tetraplegia (Gutiérrez-Ortiz et al., 2020; Toscano et al., 2020). Intubation during the acute phase of the disease may decrease neurological drive from the motor cortex to the diaphragm (Sharshar et al., 2004), and hamper extubation and recovery of normal respiration. Further, for patients living with neurological disorders, COVID-19 infection and its treatments may also exacerbate these preexisting conditions, for instance increasing disease activity symptom experience following acute systemic inflammation (Benussi et al., 2020).

There is a large and growing body of work demonstrating benefits of tES for neurorehabilitation (Brunoni et al., 2014; Cioato et al., 2016; Sasso et al., 2016; Aftanas et al., 2018; Leffa et al., 2018). In addition, tES applied as an add-on technique with cognitive or physical rehabilitation improve training outcomes, as widely demonstrated in a range of neurological conditions including post-stroke recovery (Babyar et al., 2016; Sebastian et al., 2016; Bornheim et al., 2020; Yan et al., 2020), multiple sclerosis (MS; Charvet et al., 2018; Pilloni et al., 2020), and Parkinson’s disease (Agarwal et al., 2018; Ganguly et al., 2020).

As results of the acute neurological events related to COVID-19 infection, functional outcomes of patients surviving an ICU admission report also a high prevalence of speech, language and cognitive-communication disorders (Avula et al., 2020). tDCS has been previously applied concurrently with speech therapy for language recovery in aphasia (Galletta et al., 2016; Fridriksson et al., 2018, 2019).

### Role in Managing Persistent Post-infectious Symptoms – Pain and Fatigue

More than 87% of COVID-19 patients report at least one persisting symptom 60 days following initial recovery (Carfì et al., 2020), with the most frequent being fatigue, dyspnea and pain. tES treatments have been established as a treatment approach for reducing fatigue (Lefaucheur et al., 2017), for instance in athletes (Okano et al., 2015) and in neuroimmune conditions such as multiple sclerosis (Charvet et al., 2018) and post-polio syndrome (Acler et al., 2013), possibly due to the restoration of autonomic imbalance (Chaudhuri and Behan, 2004; Louati and Berenbaum, 2015; Okano et al., 2015). The efficacy of tES for pain management has also been well-characterized (David et al., 2018; Baptista et al., 2019), including specifically reducing musculoskeletal pain [e.g., in fibromyalgia and arthralgia (Castillo-Saavedra et al., 2016; Silva-Filho et al., 2018)]. tDCS can also reduce pain (Staud, 2008; Miglis, 2018) and is under investigation for treating dyspnea (see Table 1).

**TABLE 1:** Ongoing and completed research studies across the four intervention pathways.

**COVID-19 intervention pathways**	**Aim of the trial**	**Stimulation protocol/Target area**	**Status (Reference/Clinical trial ID number)**
Acute intervention (1)	Sham-controlled RCT evaluating efficacy of tDCS on dyspnea in patients admitted to ICU	Low intensity anodal and cathodal tDCS (30 min) Primary motor cortex	Study initiated in 2018, no specific to COVID-19 Azabou et al., 2020, NCT03640455
Managing acute and chronic psychological condition (3–4)	Case report evaluating the effect of tDCS on relieving severe anxiety in COVID-19 patient	Five sessions of anodal tDCS (20 min, 2 mA) post 9 days of ICU DLPFC (F3–F4 montage)	Case report (Study completed) Shinjo et al., 2020
Managing acute and chronic psychological condition (4)	CT evaluating the effect of at-home tDCS-Limited Total Energy (tDCS-LTE) on major depressive symptoms in in the context of COVID-19 pandemic	20 sessions of anodal tDCS (20 min, 2 mA) DLPFC (F3–F4)	Ongoing CT (Recruiting) FDA investigational device exception (IDE) clinical trial
Managing mental health (4)	Sham-controlled RCT evaluating the potential of tDCS in protecting older adults from cognitive and mental health effects during pandemic outbreaks	Anodal tDCS (20 min, 2 mA) paired with cognitive/educational training DLPFC (F3–F4)	Ongoing RCT where a COVID-19 related aim was added (NCT02851511)

### Role in Managing Acute and Chronic Psychological Conditions and for Mental Health

Perhaps the most direct application of tES in the management of COVID-19 is for mental health. To date, the most extensive evidence for tES efficacy is in the management of neuropsychiatric conditions such as anxiety, PTSD, and depression (Razza et al., 2020). For those with acute infection, and particularly with history of hospitalization and ICU stay for critical illness, there can be a “post-intensive care syndrome” as a combination of acute psychological distress, such as post-traumatic stress disorder and features of acute anxiety and/or short- and long- depression (Rogers et al., 2020). To date, there has been a case report showing that tDCS over DLPFC can be a potential and adjuvant therapy for post-COVID-19 acute anxiety (Shinjo et al., 2020).

Further, there are increasing concerns about the mental health (e.g., anxiety, mood) consequence of COVID-19 pandemic, resulting from the illness (Nguyen et al., 2020), or more generally the stressors and society (medical care) disruptions associated with the COVID-19 pandemic (Li Z. et al., 2020). tES has established efficacy to treat mental illness (Kekic et al., 2016; Sagliano et al., 2019), with potential proposed applications in mitigating the mental health consequences related to COVID-19 (Bikson et al., 2020; Castelo-Branco and Fregni, 2020; Caulfield and George, 2020).

The COVID-19 pandemic and mitigation efforts have been associated with elevated distress in general, provoking a surge of addictive behaviors, new and relapse, including misuse or abuse of alcohol, and/or drugs (Dubey et al., 2020). Particularly those with substance use disorders present immune system, respiratory and pulmonary changes that may increase susceptibility to COVID-19 infection (Dubey et al., 2020). Among the resources available to assist individual in the recovery, tES and TMS have been used in a growing number of studies for its therapeutic potential in treating substance use disorder (e.g., nicotine, alcohol, and cocaine; Coles et al., 2018; Ekhtiari et al., 2019).

General population, health-care workers, as well as patients in post-acute recovery, report high rates of sleep disorders (e.g., acute insomnia) related mainly to changes in stress levels and anxiety (Huang and Zhao, 2020). In this context, recent studies investigated using tDCS to modulate top-down control of emotion regulation with positive mediating effect on sleep (Valle et al., 2009; Zhou et al., 2020).

Importantly, tDCS has been developed for home use, with remote supervision, providing an advantage for access to treatment (Charvet et al., 2020) and ensuring continuity in the treatment (Bikson et al., 2020). In addition to the portability for remote administration, tES techniques are considered safe and well-tolerated (Bikson et al., 2016; Chhabra et al., 2020). However, even as tDCS is considered to be safe, it remains investigational for many of the potential applications in COVID-19. Trials of tES in patients with acute and/or severe illness (Riggs et al., 2018), should consult relevant literature on tES trials including in those with conditions such as myocarditis or thrombotic stroke.

Table 1 summarizes some ongoing or recently completed efforts across the four intervention pathways described:

## Conclusion

As with any medical intervention, the use of tES will depend on informed decision by the caregiver team applied at the appropriate stage relative to other treatments. Any use of tES is subject to regulatory factors and must be informed by, and responsive to, the current state of research. The COVID-19 pandemic has generated special urgency to discover and deploy new treatments. tES warrants investigation and has an extensive record of safety and tolerability across clinical studies completed to date. However, in the absence of any clinical studies in COVID-19 illness, its safety and tolerability in this specific patient population has yet to be established.

Ongoing research challenges in tES are comparable to those faced for other treatments (e.g., individual variability in response to pharmacotherapy), and these unknowns must be judicially balanced against the immediate clinical need for validated new treatments during the COVID-19 pandemic. It is not realistic for multi-center controlled pivotal trials of tES to be completed for COVID-19 applications within this urgent timeframe. However, as we review, the extensive referenceable clinical data in comparable conditions, a mechanistic basis, and emerging trials in COVID-19 subjects warrant consideration.

## Author Contributions

All authors contributed to the content of this review, edited the manuscript, and approved the submission.

## Conflict of Interest

The City University of New York holds patents on brain stimulation with MB as inventor. MB has equity in Soterix Medical Inc. MB consults, received grants, assigned inventions, and/or serves on the SAB of Boston Scientific, GlaxoSmithKline, Mecta, Halo Neuroscience, X. MG has no equity ownership in any device or pharmaceutical company. MG does occasionally consult with industry, although he has not accepted consulting fees from anyone who manufactures a TMS device, because of his role in NIH and DOD/VA studies evaluating this technology. His total industry related compensation per year is less than 10% of his total university salary. The Medical University of South Carolina holds patents on brain stimulation with BB as inventor. BB has equity in Bodhi NeuroTech, Inc. The remaining authors declare that the research was conducted in the absence of any commercial or financial relationships that could be construed as a potential conflict of interest.
